# Iron Status of Kenyan Pregnant Women after Adjusting for Inflammation Using BRINDA Regression Analysis and Other Correction Methods

**DOI:** 10.3390/nu11020420

**Published:** 2019-02-16

**Authors:** Martin N. Mwangi, Elizabeth Echoka, Marthe Knijff, Lydia Kaduka, Brenda G. Werema, Frida M. Kinya, Richard Mutisya, Erastus M. Muniu, Ayşe Y. Demir, Hans Verhoef, Raphaelle Bourdet-Sicard

**Affiliations:** 1 Division of Human Nutrition, Wageningen University and Research, P.O. Box 9101, 6700HB Wageningen, The Netherlands; knijffmarthe@gmail.com (M.K.); hans.verhoef@icloud.com (H.V.); 2 Training and Research Unit of Excellence, Department of Public Health, College of Medicine, University of Malawi, Private Bag 360, Chichiri, Blantyre 3, Malawi; 3 Centre for Public Health Research, Kenya Medical Research Institute (KEMRI), P.O. Box 54840 00200, Nairobi, Kenya; lizechokah@gmail.com (E.E.); lydia.kaduka@gmail.com (L.K.); rimusya03@gmail.com (R.M.); muniue@yahoo.com (E.M.M.); 4 Danone Nutricia Africa & Overseas, Kenrail Towers, Ring Road Parklands, Nairobi, Kenya; Brenda.WEREMA@danone.com (B.G.W.); Frida.KINYUA@danone.com (F.M.K.); 5 Laboratory for Clinical Chemistry, Meander Medical Centre, Maatweg 3, 3813 TZ Amersfoort, The Netherlands; AY.Demir@meandermc.nl; 6 Cell Biology and Immunology Group, Wageningen University and Research, P.O. Box 338, 6700AH Wageningen, The Netherlands; 7 MRC Unit, The Gambia, Atlantic Boulevard, Fajara, Republic of Gambia; 8 MRC International Nutrition Group, London School of Hygiene and Tropical Medicine, Keppel Street, London WC1E 7HT, UK; 9 Danone Nutricia Research, route de la Vauve, 91120 Palaiseau, France; Raphaelle.BOURDET-SICARD@danone.com

**Keywords:** acute-phase proteins, C-reactive protein, α_1_-acid glycoprotein, ferritin, inflammation, Kenya, pregnant women

## Abstract

Serum ferritin concentration is the preferred biomarker to assess population iron status in the absence of inflammation. Interpretation of this biomarker is complicated in populations with a high burden of infection, however, because inflammation increases serum ferritin concentration independently of iron status. We aimed to compare estimates of iron status of Kenyan pregnant women, with circulating ferritin concentrations adjusted for inflammation using newly proposed methods by the BRINDA project, or using previously proposed adjustment methods. We re-analyzed data from pregnant Kenyan women living in a rural area where malaria is highly endemic (*n* = 470) or in an urban area (*n* = 402). As proposed by the BRINDA group, we adjusted individual ferritin concentration by internal regression for circulating concentrations of C-reactive protein (CRP) and *α*_1_-acid glycoprotein (AGP). Other adjustment methods comprised: (a) arithmetic correction factors based on CRP or AGP; (b) exclusion of subjects with inflammation (CRP >5 mg/L or AGP >1 g/L); and (c) higher ferritin cut-off value (<30 μg/L). We additionally adjusted for *Plasmodium* infection as appropriate. Lastly, we assessed iron status without adjustment for inflammation. All correction methods increased prevalence of iron deficiency compared to the unadjusted estimates. This increase was more pronounced with the internal regression correction method. The iron deficiency prevalence estimate increased from 53% to 87% in rural Kisumu study and from 30% to 41% in the urban Nairobi study after adjusting for inflammation (CRP and AGP) using the BRINDA internal regression method. When we corrected for both inflammation and *Plasmodium* infection using the regression correction, it resulted in lower prevalence estimates compared to uninfected women. Application of linear regression methods to adjust circulating ferritin concentration for inflammation leads to markedly decreased point estimates for ferritin concentration and increased estimates for the prevalence of iron deficiency in pregnancy.

## 1. Introduction

Iron deficiency is one of the most widespread nutritional deficiencies worldwide. Despite its recognition as a public health concern, there is a lack of reliable prevalence data globally and regionally [1]. Serum ferritin concentration, an indicator of body iron stores, is recommended by WHO to assess population iron status in the absence of inflammation [2], but it also increases during inflammation, independently of iron status. Thus, when not taking inflammation into account, the prevalence of iron deficiency is underestimated in populations with a high burden of infections [3]. Iron deficiency in pregnancy is associated with adverse health outcomes for both mother and infant, such as maternal mortality, preterm birth, being born small for gestational age, and low birth weight [4]. Accurate assessment of iron deficiency is necessary to evaluate nutrition interventions in pregnancy and infancy.

Several methods have been proposed to assess iron status in the presence of inflammation. Scientists from the Biomarkers Reflecting Inflammation and Nutrition Determinants of Anemia (BRINDA) project recently proposed the use of linear regression methods to adjust ferritin concentration for the effects of inflammation [5]. Previously proposed methods to assess iron status are: (a) correction factors for ferritin concentration [2,6,7]; (b) exclusion of subjects with inflammation from the analysis; and (c) increased cut-off values for serum ferritin concentration to define iron deficiency. All of these methods rely on concurrent inflammatory markers. C-reactive protein (CRP) is an acute phase protein that increases rapidly after the onset of inflammation and declines rapidly after cessation of inflammatory stimuli. By contrast, *α*_1_-acid glycoprotein (AGP) increases and declines slowly with these events. 

To our knowledge, although the regression correction method proposed by the BRINDA group has been tested in children and women of reproductive age, no data have been reported so far on prevalence estimates of iron deficiency in pregnancy using this method. In this paper, we re-analyzed two datasets of Kenyan pregnant women—urban and rural—to assess to what extent this newly developed regression method adjusting for inflammation will change the assessment of ferritin concentrations and the prevalence of iron deficiency in pregnancy.

## 2. Materials and Methods 

Study design and population: We used data from studies that were conducted from 2011 to 2014 in two distinct regions in Kenya—a rural area (Kisumu County, Kisumu, Kenya; henceforth referred to as the Kisumu data), and an urban area (Nairobi County, Nairobi, Kenya; henceforth referred to as the Nairobi data). *Plasmodium* infection is highly endemic in the Kisumu study area but is uncommon in Nairobi study area due to its high altitude (around 1,650–1,800 m above sea level). In Nairobi, *Plasmodium* infection is mostly imported from other regions. The main results of these studies have been published elsewhere [8,9].

Rural area (Kisumu) data: The prenatal iron and malaria (PIMAL) study concerned a randomized placebo-controlled trial to measure the effect of antenatal iron supplementation on maternal *Plasmodium* infection risk, and maternal and neonatal outcomes at delivery and one-month post-partum. For the current study, we used data from samples collected at baseline. The study was conducted in the administrative areas of Ojolla, Kanyawegi, Osiri, and Rota Sub-Locations, Kisumu County, Kisumu, Kenya. Pregnant women were recruited when aged 15–45 years, with singleton pregnancies, gestational age 13–23 weeks (determined by obstetric ultrasonography), and hemoglobin concentration >90 g/L. Venous blood samples were collected in EDTA tubes. Plasma concentrations of ferritin, soluble transferrin receptor, transferrin, CRP, and AGP were assessed on a Beckman Coulter Unicel DxC 880i analyzer at Meander Medical Centre, Amersfoort, The Netherlands [9]. For CRP, data below the assay limit of detection (LOD) of 1 mg/L were censored and reported by the laboratory as imputed values at LOD/2 (i.e., 0.5 mg/L). *Plasmodium* infection was indicated by the presence in plasma of *Plasmodium* antigens (histidine-rich protein-2, which is specific for *P*. *falciparum*; or lactate dehydrogenase specific to either *P*. *falciparum* or to non-falciparum human *Plasmodium* species; Access Bio rapid dipstick test), or the presence in erythrocytes of *P*. *falciparum*-specific DNA, as determined by quantitative polymerase chain reaction.

Urban area (Nairobi) data: The MNS 2014 study concerned a survey to assess micronutrient status, nutritional knowledge, and dietary patterns among pregnant women in their second trimester of pregnancy who attended antenatal clinics at Aga Khan Hospital, St. Mary’s Hospital, and Mama Lucy hospital in Nairobi County, Kenya [8]. The three hospitals were purposely chosen to represent urban women from high, medium, and low socio-economic status, respectively. The subjects recruited were sampled consecutively and proportionately to the daily turnover of women in their second pregnancy trimester for each of these three facilities, until the sample size for each facility was attained. Experienced research staff were trained on study-specific procedures of data collection, specimen handling, and analysis. Venous blood was collected in EDTA tubes. *Plasmodium* infection tests were done on site using rapid diagnostic tests specific for *P*. *falciparum* (histidine-rich protein 2). Serum concentrations of ferritin, soluble transferrin receptor, CRP, and AGP were measured by a multiplex enzyme immunoassay sandwich method with fluorescence detection [10]. No limit of detection was reported for CRP.

Sample size requirements were calculated for the original purposes of each study and not reported because they are irrelevant to the present article.

### 2.1. Ethics and Registration

The PIMAL study was approved by independent ethics committees from London School of Hygiene and Tropical Medicine, UK, and the Kenyatta National Hospital/University of Nairobi, Kenya. It was registered at Clinicaltrials.gov (identifier: NCT 01308112). For re-analysis of the Kisumu data for the current article, the authors obtained additional approval from the Kenyatta National Hospital/University of Nairobi Ethical Review Board. The MNS 2014 study was approved by the Kenya Medical Research Institute Scientific and Ethics Review Unit (KEMRI/CPHR/SERU/2769—www.kemri.org) and the Aga Khan University Research Ethics Committee (2014/REC-53). Written informed consent was obtained from all study subjects in both studies. 

### 2.2. Statistical Analysis

The following data, collected in the second pregnancy trimester, were used for this article: Kisumu 2011–2013 data and Nairobi 2014 data. Statistical Package for Social Sciences (SPSS) software version 22 and SAS 9.4 software (SAS Institute, Cary, NC, USA) were used for data analysis. 

As per recommendations by the BRINDA group, we used the Internal Regression Correction (IRC) approach (10) to adjust for inflammation using CRP and AGP. The IRC approach uses linear regression to adjust a biomarker by the concentration of CRP or AGP on a continuous scale and *Plasmodium* infection as a dichotomous variable. Ferritin concentration was log-transformed to normalize its distribution and to stabilize its variance, and concentrations of CRP and AGP concentrations were log-transformed under the assumption that this would linearize their relationship with the log-transformed ferritin concentration. Thus, the following regression equation was applied to adjust individual ferritin concentrations:

ln(*ferritin_adj_*) = ln(*ferritin_unadj_*) − β_1_[ln(*CRP_obs_*) − ln(*CRP_ref_*)] − β_2_[ln(*AGP_ob_*_s_) − ln(*AGP_ref_*)] − β_3_(*Plasmodium* infection) (1)

where the subscripts *adj* and *unadj* refer to adjusted and unadjusted ferritin concentrations, β_1_, β_2_, and β_3_ are the regression coefficients for CRP, AGP, and *Plasmodium* infection, respectively, and the subscript *ref* refers to reference values that are recommended under the assumption that ferritin concentrations increase only when these inflammatory markers exceed this threshold value [5,11]. For CRP, internal reference values employed were 0.5 mg/L and 1.0 mg/L for Kisumu and Nairobi, respectively. For AGP, internal reference values utilized were 0.5 g/L and 0.3 g/L for Kisumu and Nairobi, respectively. A test of multicollinearity between log-transformed CRP and AGP (ln-CRP and ln-AGP) was assessed on the basis of a test of tolerance (>0.1) to determine whether it was appropriate to include all variables in the model. Because the BRINDA group did not report estimates for the regression coefficients for their meta-regression of data from pregnant women, we estimated these coefficients separately for the Kisumu and the Nairobi studies. Estimates for the regression coefficients were exponentiated to express associations in the original units of measurements. Iron deficiency was determined by applying a cut-off of <15 μg/L [2] to inflammation-corrected ferritin concentrations.

As per BRINDA recommendations, the lowest deciles of CRP and AGP were set as internal reference values to avoid over-adjustment for low levels of inflammation, and adjustments were restricted to ferritin concentrations that corresponded to the CRP or AGP exceeding their lowest decile. Specific internal reference values were obtained for each of the groups described above. This was done on the basis of all values for CRP and AGP, as was suggested by the BRINDA group. We considered, however, that regression over censored independent variables is likely to result in biased estimators for the regression coefficients. For this reason, we also conducted an analysis of the Kisumu dataset with exclusion of observations with CRP values below the LOD. Although this truncation leads to a reduced sample size, and thus to a loss of efficiency, it has the advantages that the method is simple and that regression coefficients will be consistent, i.e., with increasing sample size, the estimates will tend towards the true value [12].

The categorical Correction Factor (CF) approach, as proposed by Thurnham et al. [6], uses arithmetic CFs that are derived from the following 4-group inflammation-adjustment model: (1) reference (CRP concentration ≤5 mg/L and AGP concentration ≤1 g/L); (2) incubation (CRP concentration >5 mg/L and AGP concentration ≤1 g/L); (3) early convalescence (CRP concentration >5 mg/L and AGP concentration >1 g/L); and (4) late convalescence (CRP concentration ≤5 mg/L and concentration AGP >1 g/L). In addition, CFs were derived by grouping those with inflammation or *Plasmodium* infection into 2 groups, in which CRP, AGP, or *Plasmodium* infection were used independently of each other. Internal Correction Factors (ICFs) were then generated by dividing geometric mean (GM) ferritin values of the non-inflammation group by GM ferritin values of each inflammation group:

ICF = GM(ferritin*_ref_*)/GM(ferritin*_inflam_*) (2)

where *ref* and *inflam* denote the reference group and the inflammation group, respectively.

Subsequently, raw ferritin values in individuals in the groups with raised inflammatory markers were multiplied by the ICFs matching their respective inflammation group to arrive at adjusted ferritin values. In line with the IRC approach, ICFs were calculated for each of the groups described above. To compare ferritin concentrations between Kisumu and Nairobi, after excluding cases with inflammation, a *t*-test was utilized to test the log-normal ratio of the geometric means and obtain corresponding 95% CIs. 

In the “exclusion” approach, individuals with inflammation (as defined by a CRP concentration >5 mg/L or AGP concentration >1 g/L, or both) or with *Plasmodium* infection were excluded from the analysis. The estimated prevalence of iron deficiency was then calculated among those remaining. The method used to calculate 95% CIs of prevalence estimates was Wilson’s score interval.

For the increased ferritin concentration approach, we defined iron deficiency as ferritin concentrations <15 μg/L or <30 μg/L in individuals without or with inflammation (CRP concentration >5 mg/L or AGP concentration >1 g/L, or both), respectively. Wilson’s score interval was used to calculate corresponding 95% CIs.

Lastly, we reported unadjusted prevalence estimates for iron deficiency. Again, 95% CIs were obtained with Wilson’s score interval. 

## 3. Results

### 3.1. Participant Characteristics

Full data were available for 864 of 872 women who were randomized (99%) in both studies. Women from Kisumu were slightly younger than women from Nairobi (24.8 years versus 27.8 years) (Table S1).

The prevalence of inflammation, whether assessed by CRP or AGP, was higher in the Kisumu population than in the Nairobi population, which reflects the higher prevalence of Plasmodium infection (Kisumu, 37.2% versus Nairobi: 0.8%) (Table S1). As such, we restricted analysis on Plasmodium infection to the Kisumu data only. In both Kisumu and Nairobi women, elevated CRP concentrations were more common than elevated AGP concentrations (Kisumu: 44.5% versus 17.2%; Nairobi: 41.8% versus 2.5%) (Table S1).

#### 3.1.1. Correction Methods

##### Method 1: IRC Approach to Correct for Inflammation

The results of the linear regression analysis are presented in Table 1. Results obtained for all Kisumu women and for Nairobi women were identical to those obtained with the use of the SAS macro provided by the BRINDA group. There was no evidence of multicollinearity. In Kisumu women, exclusion from the analysis of women with censored CRP values (i.e., CRP values below the LOD) resulted in an increased magnitude of the slope between log-ferritin concentration and log-CRP (bivariate analysis: 0.328 versus 0.201; multivariate analysis: 0.120 versus 0.005), which indicates the bias that may occur due to inclusion of censored values for the independent variables. Such exclusion reduced the sample size by 11.1% (from 470 to 418 women). In multivariate analysis, the regression coefficient for the association between log-ferritin concentration and log-CRP were less than the values obtained in bivariate analysis (Kisumu women, excluding women with CRP < LOD: 0.328 versus 0.120; Nairobi women: 0.149 versus 0.084), which indicates that this relationship was partly captured by the inclusion of log-AGP and *Plasmodium* infection in the model. In Kisumu women with CRP values exceeding the LOD, log-transformed variables for CRP and AGP, as well as *Plasmodium* infection, were independently and strongly associated with log-ferritin concentration. The same was found in Nairobi women, indicating that all three explanatory variables must be taken into account when ferritin concentrations are adjusted for inflammation.

**Figure 1:**
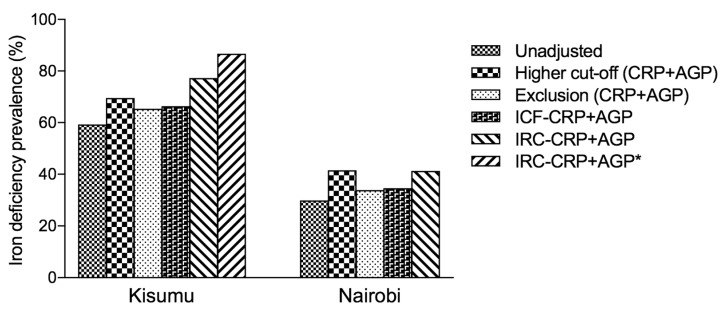
Effect of inflammation on the estimated prevalence of depleted iron stores in Kenyan pregnant women—comparison of methods of adjustment in Kenyan pregnant women, by study site, excluding women with *Plasmodium* infection. Ferritin concentrations were adjusted by various methods, based on the concentration of both C-reactive protein (CRP) and *α*_1_-acid glycoprotein (AGP). Methods based on adjustment of ferritin concentration: higher cut-off value for ferritin, depleted iron stores defined as ferritin concentration <15 µg/L in women without inflammation, and ferritin concentration <30 µg/L in women with inflammation. Exclusion: prevalence estimation restricted to women with inflammation, defined as either CRP concentration >5 mg/L or AGP concentration >1 g/L. Internal correction factor, ICF: adjustment of ferritin concentration using correction factors (Thurnham method). Internal regression correction, IRC: adjustment of ferritin concentration using linear regression analysis (BRINDA method); *: exclusion of values below limit of detection for CRP.

Compared with the unadjusted prevalence, the IRC approach resulted in increased estimates of the prevalence of iron deficiency, with highest estimates obtained when ferritin concentrations were adjusted for CRP and AGP (Figure 1, Table 2). Further increases of these estimates occurred when excluding women with *Plasmodium* infection, and when excluding women with censored CRP data. Thus, the estimated prevalence of iron deficiency in Kisumu increased from 52.8% (all women) to 86.7% (excluding cases with *Plasmodium* infection and censored CRP data, with adjustment for CRP and AGP), whilst the prevalence estimate in Nairobi increased from 29.9% to 41.3% (Table 2).

##### Method 2: ICF Approach to Correct for Inflammation

Correction factors were below 1 for all inflammatory states except for Nairobi women in the “late convalescence” state, where a correction factor of 2.96 was derived due to the small number of women in this category (Table 2, Table 3). Ferritin concentration was increased in inflammation and *Plasmodium* infection (Table 3), with highest values in women with elevated concentrations for both CRP and AGP and with *Plasmodium* infection. When excluding cases with inflammation, women in Kisumu had lower ferritin concentrations than their counterparts in Nairobi (geometric mean: 11.8 µg/L versus 22.4 µg/L; ratio, 95% CI: 0.52, 0.46 to 0.61).

The estimated prevalence of iron deficiency increased after the ICF approach was used to adjust ferritin concentrations in comparison with the unadjusted prevalence (Table 2). Thus, the estimated prevalence of iron deficiency in Kisumu increased from 52.8% (all women) to 66.4% (excluding cases with *Plasmodium* infection, with adjustment for CRP and AGP), whilst the prevalence estimate in Nairobi increased from 29.9% to 34.6%.

##### Method 3: Exclusion of Subjects with Inflammation

Exclusion of subjects with CRP > 5 mg/L or AGP > 1 g/L resulted in a sample size loss of 46.4% and 42.1% for Kisumu and Nairobi, respectively (Table 2). The estimated prevalence of iron deficiency in Kisumu increased from 52.8% (all women) to 65.4% (excluding cases with *Plasmodium* infection, with adjustment for CRP and AGP), whilst the prevalence estimate in Nairobi increased from 29.9% to 33.9%.

##### Method 4: Increased Ferritin Concentration Cut-off (<30 μg/L) in the Presence of Inflammation

The estimated prevalence of iron deficiency in Kisumu increased from 52.8% (all women) to 69.5% (excluding cases with *Plasmodium* infection, with adjustment for CRP and AGP), whilst the prevalence estimate in Nairobi increased from 29.9% to 41.5% (Table 2).

#### 3.1.2. Comparison of Methods

Figure 1 summarizes estimates of iron deficiency obtained with various methods (see preceding sections), with ferritin concentrations adjusted for both CRP and AGP, excluding cases with *Plasmodium* infection. The highest estimates were obtained with the BRINDA internal regression coefficients, even when adjusting for CRP alone or AGP alone (Figure S1).

## 4. Discussion

We aimed to compare estimates of iron status of Kenyan pregnant women, with circulating ferritin concentrations adjusted for inflammation using newly proposed methods by the BRINDA project, or using previously proposed adjustment methods. Application of linear regression methods to adjust circulating ferritin concentration for inflammation led to markedly decreased point estimates for ferritin concentration and increased estimates for the prevalence of iron deficiency. We observed better (higher) estimates of the prevalence of iron deficiency after correcting for inflammation using the BRINDA (IRC) regression method compared to other conventional approaches. This was similar to observations in non-pregnant populations [5].

All examined indicators of iron status were affected by inflammation. As expected, the geometric mean of ferritin was lowest in women without inflammation, probably because ferritin is a positive acute-phase protein, which is markedly elevated during states of inflammation [13]. 

*Plasmodium* infection was associated with ferritin even after controlling for CRP and AGP (Figure S2). The criterion to compare and evaluate models is not to judge on their ability to increase estimates of the prevalence of iron deficiency, but rather to what extent they improve model fit (i.e., the ability to predict values as close as possible to the ones observed). When comparing nested models, the difference in model fit is indicated by the change in R^2^ and corresponding *p*-value. For models that differ in the presence or absence of a single explanatory variable, the *p*-value for the change in R^2^ is identical to the *p*-value that corresponds to the regression coefficient for that explanatory variable (in this case, *Plasmodium* infection). This is the reason why we believe that a model with CRP/AGP/*Plasmodium* is better than a similar model that excludes *Plasmodium*. Ignoring censored data resulted in substantial bias in the estimates of iron deficiency.

The correction of iron-status indicators for inflammation with the use of regression correction has been found to substantially change estimates of iron deficiency prevalence in both low and high infection burden settings [14]. Using AGP, on its own or in combination with CRP, led to higher prevalence estimates of iron deficiency (Figure 1 and Table 3). In our analysis, although the prevalence of iron deficiency was much higher after using the ICF approach, exclusion approach [5] or higher cut-off compared to the unadjusted estimates, we found the prevalence of iron deficiency to be highest after using the IRC-CRP+AGP approach and was highest in Kisumu. However, for the exclusion approach, the greatest absolute change in the estimated prevalence of iron deficiency in subjects with CRP ≤ 5 mg/L was observed in pregnant women from Kisumu. A similar observation in the same population was made in subjects with AGP ≤ 1 g/L. Overall, the prevalence of iron deficiency increased in both Kisumu and Nairobi groups after using the *IRC* approach to adjust for CRP or AGP.

Iron status indicators changed at low concentrations of CRP and AGP. This observation may suggest that continuous correction using a regression correction approach may better account for the full range and severity of inflammation than would the exclusion or correction-factor approaches that rely on dichotomous cutoffs to define inflammation [14]. The higher ferritin cut-off approach performed quite well with CRP in comparison with the IRC approach, though the latter was more consistent in its performance, meaning that it always gave the highest prevalence estimates of iron deficiency. In similar resource-poor, high-infection burden regions, CRP alone may be sufficient to adjust for inflammation when using the IRC approach, but there is value in using AGP whenever possible, as it does lead to higher estimates of the prevalence of iron deficiency. In addition, the exclusion approach led to a decrease in precision and may have introduced bias. The ICF approach as outlined by Thurnham et al. [6] led to lower estimates of iron deficiency prevalence than the IRC approach. We postulate that the ICF approach categories as currently defined may result in lower estimates of iron deficiency. For example, elevated CRP and AGP are now labelled as early convalescence but they also coexist in chronic infections such as *Plasmodium* infection. “Convalescence” suggests that a person is recovering from illness, but asymptomatic infections can also cause elevated CRP or AGP concentrations [6]. 

Anemia (IDA) in pregnancy is associated with adverse health outcomes for both mother and infant, such as maternal mortality, preterm birth, being born small for gestational age, and low birth weight, even if robust evidence is still lacking [15]. Assessing iron status in pregnant women is challenging for several reasons, which probably explains why there is a limited number of studies available. On top of the classical pitfalls of iron biomarkers measurement, the increase in plasma volume occurring during pregnancy leads to dilution of seric markers, such as hemoglobin and SF, and specific cut-offs need to be developed for this population. In addition, hepcidin, the master regulator for iron absorption, is suppressed during the two last trimesters of healthy pregnancies to both permit an increase in dietary iron absorption and mobilization of iron stores, even if a high level of inflammation may still induce hepcidin release during pregnancy [16]. Finally, the inflammatory status fluctuates during pregnancy, with pro-inflammatory status during the first and the third trimesters and anti-inflammatory during the second trimester, which could influence SF level. The analyses we have conducted here were based on women in their second trimester, so extrapolation to first or third pregnancy trimester is questionable.

To the best of our knowledge, this is the first attempt at an estimation of the prevalence of iron deficiency during pregnancy using the regression correction method proposed by the BRINDA group. 

In rural Kenya, based on the Kenya National Micronutrient Survey of 2011, the prevalence of iron deficiency among pregnant women was 45.6% [17]. Compared to the new estimates based on the BRINDA IRC−CRP+AGP method (range; 70.0%–86.7%), there is a gross underestimation of the true prevalence of iron deficiency by 24.4% to 41.1%. The Kenya Demographic and Health Survey of 2014 indicated that only 8% of women aged 15–49 years with a live birth in the last five years received iron supplements for the recommended 90 days or more [18]. To prevent anemia and other pregnancy-related complications, there is an urgent need to scale up the development and monitoring of public health programs to improve the iron status of pregnant women. It is highly likely that similar trends in the prevalence of iron deficiency are the norm rather than the exception in most low and middle-income countries. The prevalence of iron deficiency is not matched by comprehensive and effective public health mitigation programs. 

The current analysis used two comprehensive datasets on pregnant women from two diverse settings, rural and urban. When analyzing trial data, an important question (concerning the BRINDA method) is whether beta coefficients should be calculated within each intervention group, or whether they should be pooled for all groups. The source population of our datasets was quite unique in terms of inflammation and prevalence of *Plasmodium* infection. As such, specific correction factors, beta coefficients, and reference values were obtained for Kisumu and Nairobi separately, in order to take into account the differences that may exist between these groups. This increases the external validity of the findings and their general applicability. Furthermore, the datasets have a large sample size and contain high-quality laboratory analyses. The analysis also draws from primary data from two studies, each of which used a sampling scheme that was representative. Both datasets are comparable in that they applied similar laboratory methods for measuring the biomarkers of interest, focused on the second trimester of pregnancy, and were cross-sectional in their nature, but designed with a specific aim to look at iron deficiency prevalence in pregnancy. This comparability provides for a generalizable interpretation of the findings. 

We have presented results of analyses of data from two markedly different populations (rural versus urban). Our data was selected on the basis of convenience (i.e., the availability of two pregnant women data sets), and was cross-sectional in nature. Because the PIMAL study was a randomized controlled trial with a placebo arm, we applied the hemoglobin cut-off of >90g/L to avoid recruiting women who would need medical intervention into the placebo arm of the trial. The application of this exclusion criterion may mean that the revised estimates of iron deficiency in Kisumu may be an under-estimate of the true prevalence. We did not have a gold-standard measure of iron status to compare against (for example bone marrow iron). Because of this, it is not clear whether CRP and AGP completely explain the relationship between ferritin and inflammation or, alternatively, whether the adjustment approaches over-adjust ferritin concentrations on the basis of a third unknown confounder. A comparison of adjustment approaches against a reference standard and the use of longitudinal data would further contribute to the evidence [19]. In the absence of such a reference standard, the linear regression method is probably the best method currently available. This observation shows the need to conduct micronutrient surveys in a harmonized fashion and the need to undertake longitudinal studies [11]. 

## 5. Conclusions

Our analyses confirmed that the BRINDA approach leads to better estimates of iron deficiency during pregnancy and presents a way to correct for inflammation. The prevalence of iron deficiency may be underestimated if it is not adjusted for inflammation, particularly among pregnant women in areas with a high prevalence of inflammation or infections [20]. Because iron deficiency is of public health significance in many countries, future studies or surveys should collect enough biomarker data to enable robust analyses. Finally, our findings reflect the need for further development and validation of approaches for estimating the true prevalence of iron deficiency, especially in resource-poor high infection burden regions.

## Figures and Tables

**Table 1:** Associations between ln(ferritin) concentrations and inflammation markers, by study population (bivariate and multivariate linear regressions).

Population	*n*	Intercept	Ln(CRP, mg/L)	Ln(AGP, g/L)	*Plasmodium* Infection
Bivariate analysis					
Kisumu					
All women	470	2.481 ± 0.063	0.201 ± 0.032	NA	NA
Excluding women with CRP < LOD	418	2.203 ± 0.087	0.328 ± 0.043	NA	NA
Nairobi	402	3.000 ± 0.064	0.149 ± 0.037	NA	NA
Kisumu					
All women	470	3.105 ± 0.052	NA	1.073 ± 0.116	NA
Nairobi	402	3.615 ± 0.095	NA	0.510 ± 0.107	NA
Multivariate analysis					
Kisumu					
All women	470	2.898 ± 0.105	0.005 ± 0.040	0.908 ± 0.148	0.401 ± 0.081
Excluding women with CRP < LOD	418	2.622 ± 0.132	0.120 ± 0.052	0.783 ± 0.162	0.408 ± 0.086
Nairobi	402	3.404 ± 0.142	0.084 ± 0.042	0.083 ± 0.042	NA

Values indicate point estimates with corresponding SE. AGP: alpha-1-acid glycoprotein, expressed in mg/L; CRP: C-reactive protein, expressed in mg/L; LOD: limit of detection for CRP (1 mg/L) in the Kisumu study; NA: not applicable. CRP and AGP were entered in the models as log_e_-transformed covariates. In the Nairobi study, there was no limit of detection reported for CRP. Regression coefficients (except for the intercept) indicate the change in ln(ferritin) associated with a change in ln(CRP) or ln(AGP) by 1 unit, or the change in ln(ferritin) that is associated with *Plasmodium* infection, given that all other variables are kept constant. Exponentiation of point estimates yields the ratio of geometric mean ferritin concentration, expressed in µg/L, that is associated with these changes.

**Table 2:** Effect on the prevalence of depleted iron stores of various methods to adjust ferritin concentration for inflammation, by study population ^1^.

Adjustment Method	Kisumu				Nairobi	
	All Women		Without *Plasmodium* Infection			
	*n*	Prevalence	*n*	Prevalence	*n*	Prevalence
Unadjusted	470	52.8% (48.3%, 57.2%)	295	59.3% (53.6%, 64.8%)	402	29.9% (25.6%, 34.5%)
BRINDA internal regression method						
Including observations with CRP < LOD						
IRC-CRP	470	66.6%	295	74.2%	402	38.3%
IRC-AGP	470	68.7%	295	76.6%	402	40.1%
IRC-CRP+AGP	470	70.0%	295	77.3%	402	41.3%
IRC-CRP+AGP+*Plasmodium* infection	470	61.9%		NA		NA
Excluding observations with CRP < LOD						
IRC-CRP	418	79.2%	255	87.5%		NA
IRC-CRP+AGP	418	78.7%	255	86.7%		NA
IRC-CRP+AGP+*Plasmodium* infection	470	65.8%		NA		NA
Internal correction factor (ICF) method						
ICF-CRP ^3^	470	59.8%	295	65.8%	402	34.8%
ICF-AGP ^3^	470	56.2%	295	62.7%	402	30.4%
ICF-CRP+AGP ^3^	470	60.4%	295	66.4%	402	34.6%
Analysis restricted to women without inflammation, with inflammation defined as:						
CRP concentration >5 mg/L	261	62.1% (56.1%, 67.7%)	190	64.7% (57.7%, 71.2%)	242	36.4% (30.6%, 42.6%)
AGP concentration >1 g/L	389	57.3% (52.4%, 62.2%)	260	61.5% (55.5%, 67.2%)	400	31.5% (27.1%, 36.2%)
CRP concentration >5 mg/L or AGP concentration >1 g/L	252	63.1% (57.0%, 68.8%)	185	65.4% (58.3%, 71.9%)	233	33.9% (28.1%, 40.2%)
Iron deficiency defined as ferritin concentration <30 µg/L in women with inflammation ^2^, with inflammation defined as:						
CRP concentration >5 mg/L	470	62.1% (57.7%, 66.4%)	295	68.5% (63.0%, 73.5%)	402	41.5% (36.8%, 46.4%)
AGP concentration >1 g/L	470	56.2% (51.7%, 60.6%)	295	62.7% (57.1%, 68.0%)	402	30.4% (26.1%, 35.0%)
CRP concentration >5 mg/L or AGP concentration >1 g/L	470	63.0% (58.5%, 67.2%	295	69.5% (64.0%, 74.5%)	402	41.5% (36.8%, 46.4%)

Values indicate prevalence, % (95% CI, where applicable); 95% CIs are not reported for the BRINDA internal regression method and the internal correction factor method because they do not take into account the variability in the estimates for the regression coefficients of the slopes used to derive the adjusted ferritin values. AGP: *α*_1_-acid glycoprotein. CRP: C-reactive protein. LOD: limit of detection. NA: not applicable. Depleted iron stores were defined as ^1^ ferritin concentration <15 µg/L for all subjects, or ^2^ ferritin concentration <15 µg/L in women without inflammation and ferritin concentration <30 µg/L in women with inflammation, respectively. ^3^ Internal correction factors for Kisumu: ICF−CRP = 0.60, ICF−AGP = 0.49, ICF−CRP+AGP = 0.71 (incubation), 0.41 (early convalescence), 0.57 (late convalescence); for Nairobi 2014: ICF−CRP = 0.80, ICF−AGP = 0.49, ICF−CRP+AGP = 0.84 (incubation), 0.37 (early convalescence), 2.96 (late convalescence).

**Table 3:** Circulating ferritin concentrations in pregnant women by study site, by Plasmodium status and inflammation status.

	*Plasmodium* Infection Absent			*Plasmodium* Infection Present		
Inflammatory Stage	*n*	Ferritin, μg/L		*n*	Ferritin, μg/L	
Kisumu						
All women	295	13.1	(12.0, 14.2)	175	23.1	(19.8, 26.9)
By inflammation status						
Reference ^1^	185	11.8	(10.6, 13.0)	67	15.4	(12.7, 18.7)
Incubation	75	14.3	(12.0, 17.2)	62	23.3	(18.0, 30.1)
Early convalescence	30	19.8	(14.5, 27.1)	42	41.4	(29.4, 58.2)
Late convalescence	5	12.9	(8.0, 20.8)	4	37.7	(3.8, 378.2)
Nairobi						
All women	398	24.5	(22.6, 26.5)	2	54.5	^2^
By inflammation status						
Reference ^1^	229	22.4	(20.3, 24.7)	2	54.5	^2^
Incubation	159	26.7	(23.7, 30.2)	0	-	
Early convalescence	9	60.4	(22.4, 162.5)	0	-	
Late convalescence	1	7.6	^2^	0	-	

Values indicate geometric mean (95% CI) in µg/L. ^1^ Reference: C-reactive protein (CRP) concentration ≤5 mg/L and *α*_1_-acid glycoprotein (AGP) concentration ≤1 g/L; incubation: CRP concentration >5 mg/L and AGP concentration ≤1 g/L; early convalescence: CRP concentration >5 mg/L and AGP concentration >1 g/L; late convalescence: AGP concentration >1 g/L and CRP concentration ≤5 mg/L. ^2^ A 95% CI was not reported due to the small numbers.

## Supplementary Materials

The following are available online at https://www.mdpi.com/2072-6643/11/2/420/s1, Figure S1: Effect of inflammation on the estimated prevalence of depleted iron stores in Kenyan pregnant women: comparison of methods of adjustment in Kenyan pregnant women, by study site, excluding women with *Plasmodium* infection, Figure S2: Effect of inflammation and *Plasmodium* infection on the estimated prevalence of depleted iron stores in Kenyan pregnant women from Kisumu: comparison of methods of adjustment in Kenyan pregnant women from Kisumu, Table S1: Population characteristics of pregnant Kenyan women from Kisumu and Nairobi.
