# Supplementary material for: Iron Status of Kenyan Pregnant Women after Adjusting for Inflammation Using BRINDA Regression Analysis and Other Correction Methods
Source: Nutrients. 2019 Feb 16;11(2):420. doi: 10.3390/nu11020420 (PMC6413054; doi:10.3390/nu11020420)
Supplement: Supplementary file 1 [file nutrients-11-00420-s001.pdf]

Panel A

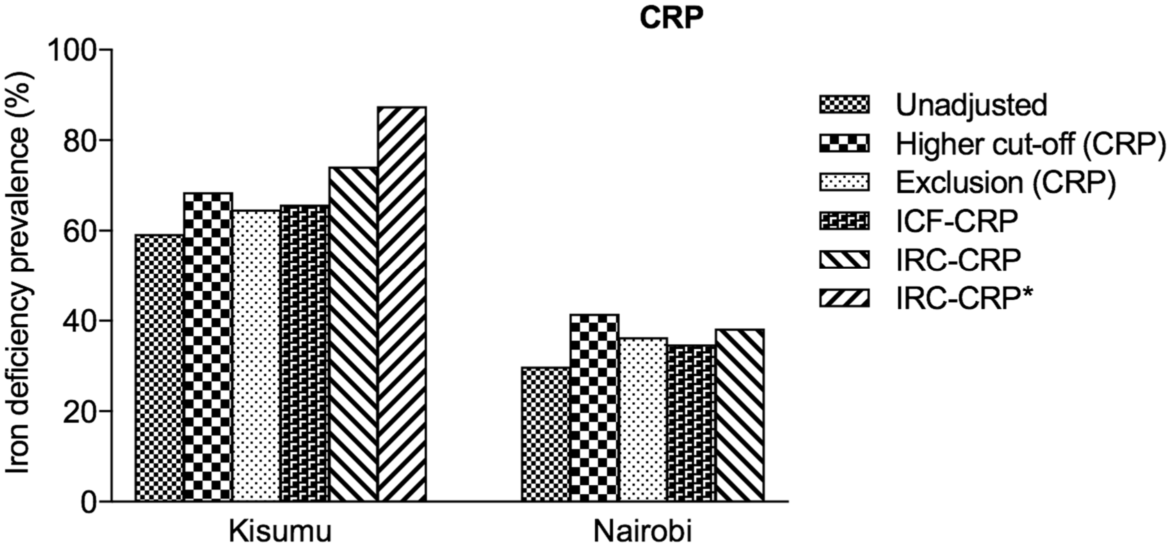

Panel B

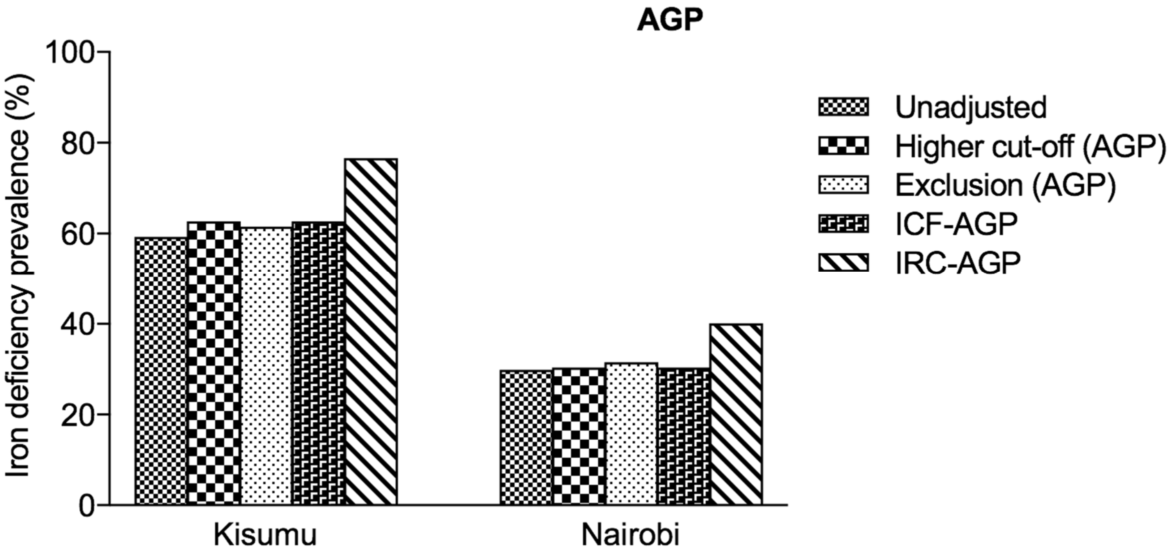

Figure S1. Effect of inflammation on the estimated prevalence of depleted iron stores in Kenyan pregnant women: comparison of methods of adjustment in Kenyan pregnant women, by study site, excluding women with *Plasmodium* infection. Ferritin concentrations were adjusted by various methods, based on the concentration of C-reactive protein (CRP) only (***Panel A***) or α_1-acid glycoprotein (AGP) only (***Panel B***). Methods based on adjustment of ferritin concentration: *higher cut-off value for ferritin:* depleted iron stores defined as ferritin concentration <15 µg/L in women without inflammation, and ferritin concentration <30 µg/L in women with inflammation; *exclusion:* prevalence estimation restricted to women with inflammation, defined as CRP concentration >5 mg/L or AGP concentration >1 g/L; *internal correction factor, ICF:* adjustment of ferritin concentration using correction factors (Thurnham method); *internal regression correction, IRC:* adjustment of ferritin concentration using linear regression analysis (BRINDA method); *: exclusion of values below limit of detection for CRP.

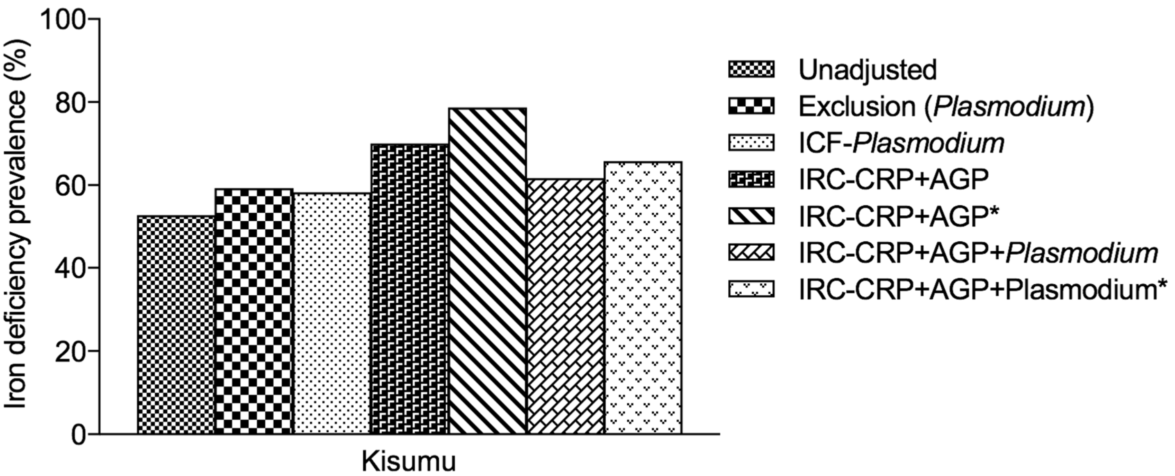

Figure S2. Effect of inflammation and *Plasmodium* infection on the estimated prevalence of depleted iron stores in Kenyan pregnant women from Kisumu: comparison of methods of adjustment in Kenyan pregnant women from Kisumu. Ferritin concentrations were adjusted by various methods, based on *Plasmodium* infection status and/or concentration of C-reactive protein (CRP) and α_1-acid glycoprotein (AGP). Methods based on adjustment of ferritin concentration: *exclusion:* prevalence estimation restricted to women with inflammation, defined as positive *Plasmodium* infection; *internal correction factor, ICF:* adjustment of ferritin concentration using correction factors (Thurnham method); *internal regression correction, IRC:* adjustment of ferritin concentration using linear regression analysis (BRINDA method); *: exclusion of values below limit of detection for CRP.

Table 1. Population characteristics of pregnant Kenyan women from Kisumu and Nairobi.

Characteristic	Kisumu		Nairobi	
n	470		410	
Missing[1]	0	(0.0)	10	(2.4)
Age, years (range)	24.8	(15.0-44.0)	27.8	(18.0-41.0)
CRP concentration >5 mg/L	209	(44.5)	168	(41.8)
AGP concentration >1 g/L	81	(17.2)	10	(2.5)
CRP concentration >5 mg/L or AGP concentration >1 g/L	218	(46.4)	169	(42.0)
Plasmodium infection	175	(37.2)	3	(0.8)

All values are *n* (%) unless stated otherwise; AGP: α_1-acid glycoprotein; CRP: C-reactive protein. [1]For Nairobi, six participants did not have CRP, AGP and *Plasmodium* infection data; two participants did not have CRP and AGP data; two other participants did not have *Plasmodium* infection data thus, total missing data was for *n* = 10.
